# Damage response of XRCC1 at sites of DNA single strand breaks is regulated by phosphorylation and ubiquitylation after degradation of poly(ADP-ribose)

**DOI:** 10.1242/jcs.128272

**Published:** 2013-10-01

**Authors:** Leizhen Wei, Satoshi Nakajima, Ching-Lung Hsieh, Shinichiro Kanno, Mitsuko Masutani, Arthur S. Levine, Akira Yasui, Li Lan

**Affiliations:** 1 Department of Microbiology and Molecular Genetics, University of Pittsburgh School of Medicine, University of Pittsburgh, Pittsburgh, Pennsylvania, 15261, USA; 2 Division of Dynamic Proteome, Institute of Development, Aging, and Cancer, Tohoku University, Seiryomachi 4-1, Sendai 980-8575, Japan; 3 Division of Genome Stability Research, National Cancer Center Research Institute, Tsukiji 5-1-1, Tokyo, 104-0045 Japan

**Keywords:** Single strand breaks, XRCC1, Damage response, Phosphorylation, PolyADP-ribosylation, Ubiquitylation

## Abstract

Single-strand breaks (SSBs) are the most common type of oxidative DNA damage and they are related to aging and many genetic diseases. The scaffold protein for repair of SSBs, XRCC1, accumulates at sites of poly(ADP-ribose) (pAR) synthesized by PARP, but it is retained at sites of SSBs after pAR degradation. How XRCC1 responds to SSBs after pAR degradation and how this affects repair progression are not well understood. We found that XRCC1 dissociates from pAR and is translocated to sites of SSBs dependent on its BRCTII domain and the function of PARG. In addition, phosphorylation of XRCC1 is also required for the proper dissociation kinetics of XRCC1 because (1) phosphorylation sites mutated in XRCC1 (X1 pm) cause retention of XRCC1 at sites of SSB for a longer time compared to wild type XRCC1; and (2) phosphorylation of XRCC1 is required for efficient polyubiquitylation of XRCC1. Interestingly, a mutant of XRCC1, LL360/361DD, which abolishes pAR binding, shows significant upregulation of ubiquitylation, indicating that pARylation of XRCC1 prevents the poly-ubiquitylation. We also found that the dynamics of the repair proteins DNA polymerase beta, PNK, APTX, PCNA and ligase I are regulated by domains of XRCC1. In summary, the dynamic damage response of XRCC1 is regulated in a manner that depends on modifications of polyADP-ribosylation, phosphorylation and ubiquitylation in live cells.

## Introduction

Single-strand breaks (SSBs), one of the most common DNA lesions in human cells, cause transcription and replication blocks, leading to genomic instability and cell death (Cooke et al., 2003). The absence of SSB repair (SSBR) substantially reduces cell survival if recombination repair is also impaired (Bryant et al., 2005; Farmer et al., 2005), indicating that a SSB, if not repaired, leads to a double-strand break (DSB) at the replication fork. Poly(ADP-ribose) polymerase (PARP) is a nick sensor and also binds to SSBs, short gaps in duplex DNA, DSBs and other abnormal DNA structures and initiates the efficient repair of SSBs (Caldecott, 2003; Lan et al., 2004; Oei et al., 2005; Okano et al., 2003). After activation of PARP at SSBs, XRCC1 accumulates at poly(ADP-ribose) (pAR) sites (El-Khamisy et al., 2003; Lan et al., 2004; Okano et al., 2003). However, pAR is degraded by pAR glycohydrolase (PARG) immediately after its production. After degradation of pAR, XRCC1 appears to act as a molecular scaffold or matchmaker, recruiting and regulating the enzymatic components of the repair process at various stages of SSBR, but the key to retention of XRCC1 at sites of DNA damage is not known.

Repair of SSBs is effected by polymerase β (Polβ)-dependent short-patch repair or by proliferating cell nuclear antigen (PCNA)/polymerase δ/ε-dependent long-patch repair. DNA ligase IIIα (LigIIIα) and ligase I (LigI) are responsible for the ligation steps of short-patch repair and long-patch repair, respectively (Petermann et al., 2006; Tomkinson et al., 2001). XRCC1 interacts with repair proteins through different domains: Polβ interacts with the N-terminal domain (NTD); PARPs (PARP1 and PARP2) interact with the BRCT I domain, and LigIIIα interacts with the BRCT II domain of XRCC1 (Caldecott et al., 1994; Marintchev et al., 2000). Besides polyADP-ribosylation, a ubiquitous protein kinase, CK2, phosphorylates XRCC1 at several sites between the BRCT I and BRCT II domains, where polynucleotide kinase (PNK) and aprataxin (APTX) also associate with XRCC1 (Loizou et al., 2004). PNK possesses both 5′-DNA kinase and 3′-DNA phosphatase activities that generate DNA ends for DNA synthesis and ligation. A mutation in the APTX gene is responsible for the ataxia that occurs in oculomotor apraxia type 1, a human neurological disease similar to ataxia-telangiectasia (Ahel et al., 2006). It also has been shown that XRCC1 is ubiquitylated by the cytoplasmic E3 ligase CHIP (Parsons et al., 2008) and by the pAR-binding E3 ligase Iduna (Kang et al., 2011) at sites of damage, indicating that multiple modifications of XRCC1 occur in cells.

How these functional domains and modifications of XRCC1 affect the repair of SSBs has been investigated in cells. The BRCT I domain of XRCC1 is required for efficient SSBR both in G1 and S-G2 phases and for cell survival following treatment with methyl-methane sulfonate (MMS), whereas the BRCT II domain of XRCC1 is required for SSBR during the G1 phase (Taylor et al., 2000; Taylor et al., 2002). Mutations in the BRCT II domain affect the speed of repair, as measured by the comet assay, but do not affect cell survival after treatment with MMS (Taylor et al., 2000). The phosphorylation mutant of XRCC1 affects the speed of SSBR as measured by the comet assay (Loizou et al., 2004). Therefore, SSBR contains at least two XRCC1-dependent pathways, including a rapid repair throughout the interphase and a slow repair specific to the S-G2 phase (Moore et al., 2000; Taylor et al., 2002). The BRCT II domain and the phosphorylation status of XRCC1 both influence rapid repair of SSBs. It is important for replicating cells to maintain genomic stability by reducing the number of SSBs which affect transcription as well as replication, although the molecular mechanism by which XRCC1 effects repair is not well understood.

By using a laser micro-irradiation system, we have previously shown that the SSB-induced polyADP-ribosylation of proteins surrounding SSBs triggers the accumulation of XRCC1 and that the recruitment of the repair synthesis proteins Polβ and PCNA depends on XRCC1 (Lan et al., 2004). To understand how XRCC1 is retained at the sites of SSBs after pAR degradation and how SSBR is organized by XRCC1, we used a mutant XRCC1 lacking the BRCT II domain (X1w/oBII) and the XRCC1 phosphorylation mutant XRCC1 (X1 pm) to characterize the damage response of XRCC1 in the repair of SSBs. We show and discuss how XRCC1 is retained at, and dissociates from, sites of DNA damage by modifications of ubiquitylation, phosphorylation, and polyADP-ribosylation, based on the function of its BRCT domains.

## Results

### The BRCT II domain is necessary for retention of XRCC1 at sites of SSBs but not at activated poly(ADP-ribose) sites, whereas phosphorylation of XRCC1 is required for its dissociation from SSBs

**Fig. 1.**
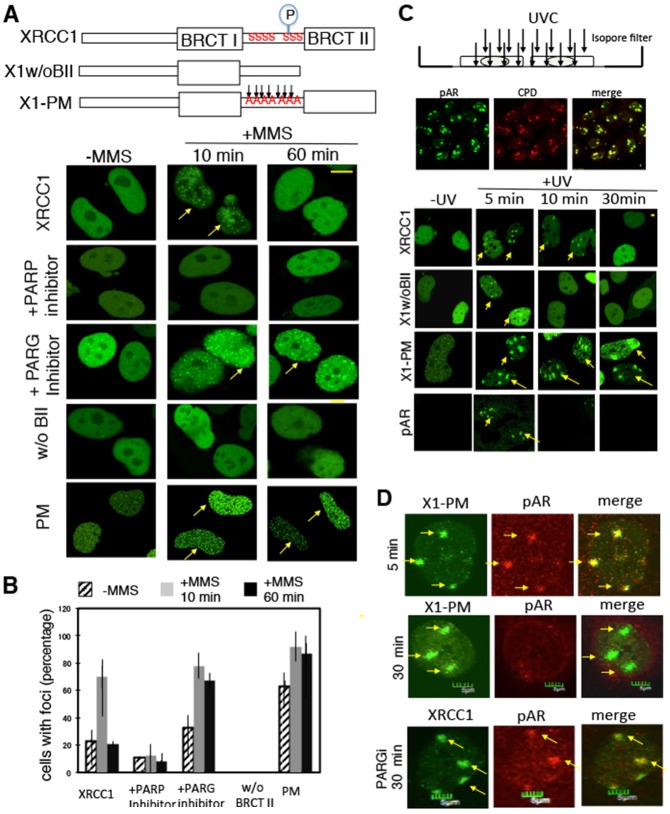
**The BRCT II domain and phosphorylation of XRCC1 are required for proper kinetics of XRCC1 at sites of damage.** (A) Top: scheme of constructs for X1w/oBII and X1 pm used in the experiments. Bottom: response of wild-type XRCC1 to treatment with 4 µM PARP inhibitor PJ34 or 100 µM PARG inhibitor tannic acid, and the response of X1w/oBII and X1 pm to 40 µg/ml MMS-induced damage in HeLa cells at the indicated times. (B) Quantification of HeLa cell fractions with MMS-induced foci before, 10 minutes, and 60 minutes after MMS treatment. (C) Scheme of SSBs induced in XPA-UVDE cells after local UVC irradiation, and damage response of XRCC1 and mutant XRCC1 to XPA-UVDE-induced SSBs after 20 J/m^2^ UVC irradiation at the indicated time points. (D) Staining of pAR in X1 pm-expressing XPA-UVDE cells 5 minutes and 30 minutes after 20 J/m^2^ UVC irradiation. Yellow arrows indicate the foci formed by XRCC1 and its mutants.

XRCC1 serves as a scaffold protein in the repair of SSBs and forms foci after methylmethane sulfonate (MMS) treatment or H_2_O_2_ treatment (El-Khamisy et al., 2003; Lan et al., 2004; Okano et al., 2003). We and other groups have shown that accumulation of XRCC1 at SSBs is dependent on PARP activation, and the BRCT I domain of XRCC1 is necessary for its accumulation in cells (Lan et al., 2004; Okano et al., 2003). However, both activation of PARP at sites of SSBs and degradation of synthesized pAR by PARG are rapid (Davidovic et al., 2001). To analyze the damage response of XRCC1 (Fig. 1A) and to understand how XRCC1 is retained at sites of SSBs after pAR degradation, we used the PARP inhibitor olaparib, PJ34, a PARG inhibitor (tannic acid) (Keil et al., 2004), a mutant XRCC1 lacking the BRCT II domain (X1w/oBII), and a mutant XRCC1 (X1 pm) harboring mutations at seven CK2 phosphorylation sites (Ser408Ala, Ser409Gly, Ser410Ala, Thr453Ala, Thr488Ala, Ser519Ala, Thr523Ala) that could not be phosphorylated by CK2 (Loizou et al., 2004). The PARP inhibitor diminished formation of foci whereas the PARG inhibitor delayed the disappearance of foci (Fig. 1A). Interestingly, we could not detect X1w/oBII-induced foci after MMS treatment, but X1 pm formed an increased number of both endogenous and MMS-induced foci (Fig. 1A), indicating that X1 pm is retained at sites of both endogenous and exogenous damage for a longer time. The cells with foci were counted and percentages are shown in Fig. 1B before and 10 minutes or 1 hour after MMS treatment. MMS-induced foci of X1 pm increased 10 minutes after MMS treatment and remained at high frequency 1 hour after MMS treatment (Fig. 1B), indicating the role of phosphorylation in XRCC1 dissociation from damage after repair.

To confirm that the kinetics of the XRCC1 mutants above are not specific to MMS treatment, another system was used to induce SSBs by expressing UV damage endonuclease (UVDE) in a human XPA cell line at the sites of local UVC irradiation (Okano et al., 2000; Okano et al., 2003). UVDE introduces nicks at sites of cyclobutane pyrimidine dimers (CPD) that XPA cells cannot repair; pAR localized with CPD at sites of SSBs (Fig. 1C). X1w/oBII dissociated from damage faster and X1 pm was retained at sites of damage longer than full-length XRCC1 (Fig. 1C). pAR could not be detected 10 minutes after UVC irradiation nor could XRCC1BII, supporting the observation that X1w/oBII dissociated from damage along with pAR (Fig. 1C). To identify whether the retention of X1 pm is due to pAR accumulation when X1 pm is expressed, we measured the pAR signals 5 minutes and 30 minutes after UV irradiation in X1 pm-expressing cells. X1 pm and pAR were colocalized 5 minutes after UV irradiation (Fig. 1D). pAR disappeared in X1 pm-expressing cells 30 minutes after irradiation, whereas X1 pm still remained at the sites of damage (Fig. 1D). In contrast, pAR was retained at the damage sites for a longer time when cells were treated with a PARG inhibitor (Fig. 1D). These data indicate that retention of X1 pm at the sites of SSBs is not mediated by delayed pAR degradation.

**Fig. 2.**
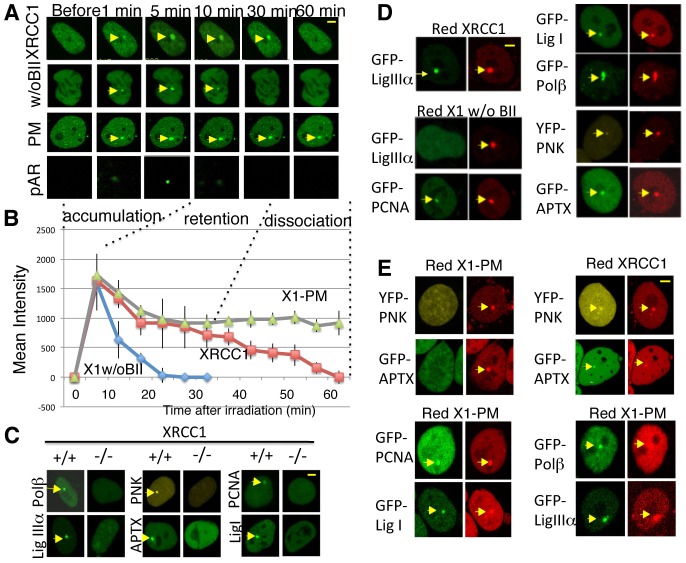
**The BRCT II domain and phosphorylation of XRCC1 are required for recruitment of repair proteins.** (A) Time-dependent accumulation of GFP-tagged full-length and mutant XRCC1 at UVA-laser-induced SSBs in HeLa cells after 405 nm laser irradiation for 10 mseconds. (B) Quantification of the intensity of foci of full-length and mutant XRCC1 in HeLa cells. (C) Accumulation of GFP–Polβ, GFP–LigIIIα, YFP–PNK and GFP–APTX in *Xrcc1*^+/+^ and *Xrcc1*^−/−^ MEF cells after 405 nm laser irradiation for 10 mseconds. There is no accumulation of each protein at SSBs in *Xrcc1*^−/−^ MEF cells. (D) The damage response of GFP–Polβ and GFP–LigIIIα, but not YFP–PNK and GFP–APTX, at sites of SSBs in *Xrcc1*^−/−^ MEF cells expressing DsRed–X1 pm after laser irradiation. (E) Damage response of GFP–Polβ, GFP–APTX, and YFP–APTX, but not LigIIIα, at SSBs in *Xrcc1*^−/−^ MEF cells expressing DsRed–XRCC1w/o BII. Yellow arrowheads show the foci formed by each indicated protein.

To follow the damage response of XRCC1 in real time, we used laser micro-irradiation through an objective lens that predominantly produces SSBs in restricted sites within a single cell nucleus in real time (Lan et al., 2004). We followed the dynamics of XRCC1 in a single cell in real time using the laser micro-irradiation system, shown in Fig. 2. From the kinetics of the damage response of XRCC1 (Fig. 2A,B), we consider that the response of full-length XRCC1 can be divided into three stages. (1) Accumulation: the very fast damage-induced recruitment of XRCC1 (within 5 minutes of PARP-synthesized pAR); (2) retention: 10–30 minutes after damage induction, pAR is degraded, but XRCC1 is still retained at the sites of damage; (3) dissociation: 30–60 minutes after damage, XRCC1 dissociates from sites of damage. The time course of XRCC1 is dependent on the dose of laser used, but the half-life of focus intensity does not change (not shown).

The mutant X1w/oBII accumulated at the damage sites but dissociated much faster than the wild-type protein (Fig. 2A,B). Twenty minutes after accumulation, the full-length XRCC1 still remained at damage sites, whereas X1w/oBII had already dissociated from SSBs. Fast dissociation of X12/oBII indicates the BRCT II domain is necessary to retain XRCC1 at the accumulated site (Fig. 1; Fig. 2A,B). In contrast to X1w/oBII, X1 pm remained at the sites of SSBs longer than wild-type XRCC1. An hour after accumulation, the intensity of full-length XRCC1 returns to the background level, indicating that it has dissociated from sites of damage, while X1 pm is still retained at SSBs (Fig. 2A,B). The kinetics of X1 pm indicates that phosphorylation of XRCC1 is not required for damage recognition, but it is for its dissociation from damage sites. Furthermore, we investigated the damage response of XRCC1 mutants in HeLa, U2OS, *Xrcc1*^+/+^, and *Xrcc1*^−/−^ MEF cell lines (50 cells each) and found kinetics with the same trends (data not shown). Therefore, the kinetics shown by X1w/oBII and X1 pm are not cell-type specific. To summarize, the BRCTII domain-deleted XRCC1 dissociated quickly from damage sites together with poly(ADP-ribose) degradation, whereas the lack of phosphorylation of XRCC1 leads to retention at sites of damage longer than wild-type XRCC1 as addressed by the three methods (MMS foci, XPA-UVDE foci, and laser micro-irradiation) we used to induce DNA SSBs.

### The BRCT II domain of XRCC1 is necessary for the damage response of LigIIIα; phosphorylation of XRCC1 is required for the damage response of PNK and APTX

To understand how BRCT II and the phosphorylation function of XRCC1 affect the damage response of other proteins, we next examined how the repair proteins are recruited to SSBs. XRCC1 interacts with various proteins involved in SSBR: Polβ interacts with the N-terminus of XRCC1, and LigIIIα interacts with the BRCT II domain of XRCC1 (Caldecott et al., 1994; Marintchev et al., 2000). PNK and APTX interact with XRCC1 through the region between the BRCT I and BRCT II domains (Loizou et al., 2004; Luo et al., 2004). Polβ, LigIIIα, PNK, APTX, PCNA and LigI all accumulated at the SSBs in *Xrcc1*^+/+^ MEF cells (Fig. 2C, left panels), but none of the proteins accumulated in *Xrcc1*^−/−^ MEF cells (Fig. 2C, right panels) or in CHO-derived XRCC1-deficient EM9 cells (not shown). Therefore, these repair proteins accumulate at SSBs in an XRCC1-dependent manner. Other XRCC1-interacting proteins, such as the glycosylases NTH1, OGG1, NEIL1 and APE1, do not accumulate at SSBs, but do accumulate at base damage induced by higher laser doses (Lan et al., 2004). The accumulation kinetics of the glycosylases NTH1, OGG1 and APE1 in *Xrcc1*^−/−^ MEF cells were the same as in *Xrcc1*^+/+^ cells (not shown), indicating that not all of the interacting proteins are recruited to XRCC1 at SSBs.

Since XRCC1 is retained at SSBs through its BRCT II domain (Fig. 1; Fig. 2A,B) and the BRCT II domain of XRCC1 interacts tightly with DNA LigIIIα (Caldecott et al., 1994), this interaction may retain XRCC1 at SSBs. We have shown that the presence of XRCC1 is essential for the accumulation of LigIIIα at SSBs (Fig. 2D). X1w/oBII complemented the accumulation of Polβ, PNK and APTX, but not LigIIIα in *Xrcc1*^−/−^ cells (Fig. 2D), indicating that the BRCT II domain of XRCC1 is necessary for recruitment of LigIIIα to sites of SSBs. To understand the influence of CK2-phosphorylation sites in XRCC1 downstream of the response to SSBs, we co-transfected *Xrcc1*^−/−^ cells with X1 pm and SSBR proteins, and examined their accumulation. In X1 pm-expressing *Xrcc1*^−/−^ cells, Polβ and LigIIIα accumulated as in wild-type cells, whereas PNK and APTX lost the damage response completely (Fig. 2E). X1w/oBII promoted the accumulation of PNK and APTX as well as wild-type XRCC1, showing that CK2-mediated phosphorylation is necessary for the recruitment of PNK and APTX to damage sites. Therefore, the BRCT II domain of XRCC1 is necessary for the damage response of LigIIIα, whereas phosphorylation of XRCC1 is required for the damage response of PNK and APTX. These results also demonstrate the importance of the interaction between XRCC1 and its partners, indicating that the damage response of repair factors in live cells is based on dynamic protein–protein interactions at sites of damage.

### The BRCT II domain but not LigIIIα is necessary to retain XRCC1 at sites of SSBs after pAR degradation

**Fig. 3.**
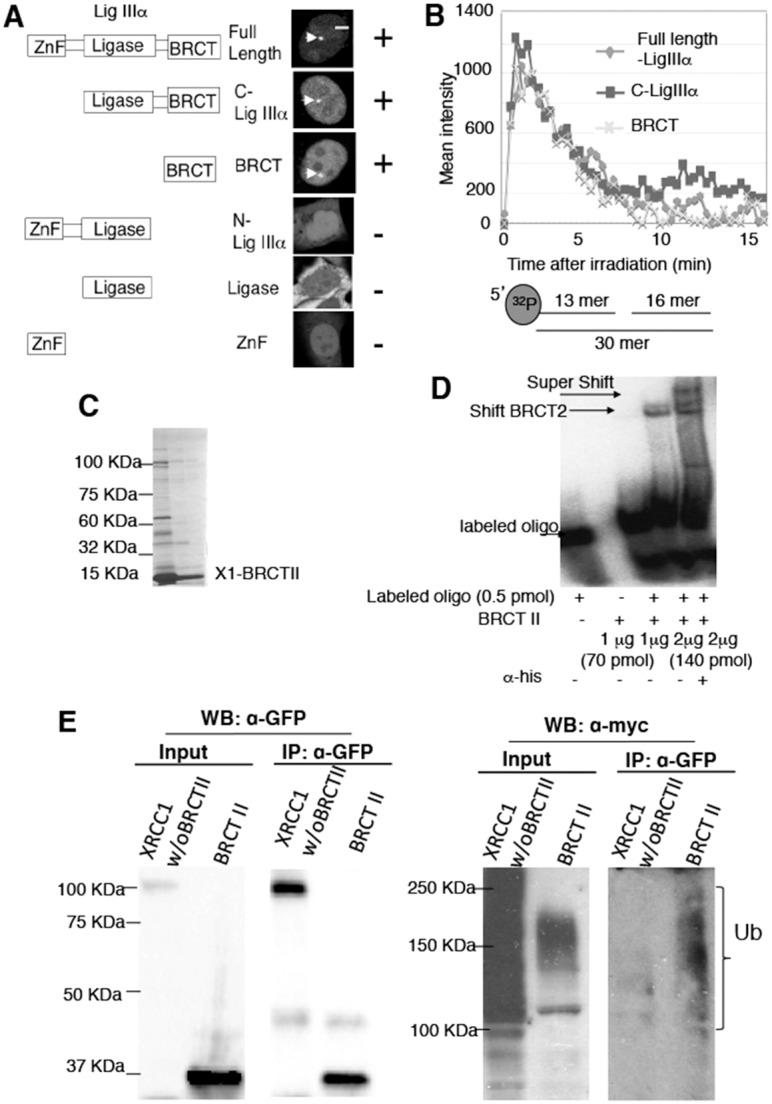
**The BRCT II domain binds nicked DNA *in vitro*.** (A) Schematic of domains and deletion mutants of LigIIIα (left) and accumulation of the deletion mutants at SSBs (right) in HeLa cells after laser irradiation. (B) Accumulation kinetics of full-length and deletion mutants of LigIIIα. (C) Purification of the XRCC1 BRCT II domain. (D) Gel shift assay with 0.5 pmol of one nucleotide gap DNA after binding with XRCC1 BRCT II domains at the indicated amounts. (E) GFP-X1w/oBRCTII or GFP-BRCTII were co-transfected with Myc-ubiquitin into HEK293 cells; the cell extracts were pulled down by anti-GFP and detected by anti-myc antibody.

Since the BRCT II domain is necessary for the recruitment of LigIIIα to sites of SSBs, we investigated whether LigIIIα plays a role in the retention of the XRCC1–LigIIIα complex at SSBs. However, siRNA treatment of LigIIIα did not affect the kinetics of XRCC1 (supplementary material Fig. S1). We further analyzed the accumulation and retention of LigIIIα deletion mutants. LigIIIα is roughly divided into three domains: an N-terminal zinc-finger (Zn-finger) domain, a central ligase domain (Ligase), and a C-terminal BRCT domain. The Zn-finger and Ligase domains of LigIIIα did not accumulate at SSBs, but the C-terminal BRCT domain did accumulate (Fig. 3A), consistent with previous data (Mortusewicz et al., 2006). The effects of these domains on kinetics were examined; neither the loss of the Zn finger nor the ligase domain affected the accumulation kinetics of LigIIIα (Fig. 3B). Therefore, the interaction of the BRCT II domain with XRCC1 enables LigIIIα to accumulate and to be retained at SSBs, but LigIIIα does not determine the retention of the XRCC1–LigIIIα complex. In addition, in XRCC1-deficient EM9 cells, Polβ dissociates from sites of SSBs when X1w/oBII is expressed faster than when full-length XRCC1 is expressed (supplementary material Fig. S2), indicating that the BRCT II domain of XRCC1 contributes to the retention of repair enzymes of SSBs at sites of damage.

As LigIIIα is not the agent of retention of XRCC1 at sites of damage, we assumed that the BRCT II domain has an affinity for nicked DNA once it is located adjacent to sites of damage. It is known that full-length XRCC1 binds nicked DNA much more efficiently than intact DNA duplexes (Mani et al., 2004; Nazarkina et al., 2007). Therefore, we purified the BRCT II domain of XRCC1 and performed a gel shift assay with a substrate labeled with ^32^P, containing one nucleotide nick. The purified BRCT II domain (Fig. 3C) showed an affinity for nicked DNA; the binding ability increased with the addition of increased concentrations of protein (Fig. 3D). A super shift was detected by adding anti-His antibody, indicating that the BRCT II domain of XRCC1 has the affinity for nicked DNA (Fig. 3D).

XRCC1 is ubiquitylated by the E3 ligase CHIP *in vitro* (Parsons et al., 2008) and Induna (RNF146) (Kang et al., 2011). The purified BRCT II domain is targeted for ubiquitylation *in vitro* (Parsons et al., 2008). To exclude the possibility that X1w/oBII is degraded rapidly and therefore dissociated from SSBs, we analyzed the ubiquitylation of X1w/oBII in cells. Ubiquitin was detected when the BRCT II domain of XRCC1 was pulled down but not when X1w/o BII was pulled down (Fig. 3E). This result confirms that ubiquitylation of XRCC1 is mediated through its BRCT II domain in cells, and that the dissociation of XRCC1w/oBII is not through ubiquitylation-mediated degradation. We concluded that it is the BRCT II domain but not LigIIIα that is necessary to retain XRCC1 at sites of SSBs after pAR degradation.

### The function of PARG is to promote attachment of XRCC1 and LigIIIα to broken DNA

**Fig. 4.**
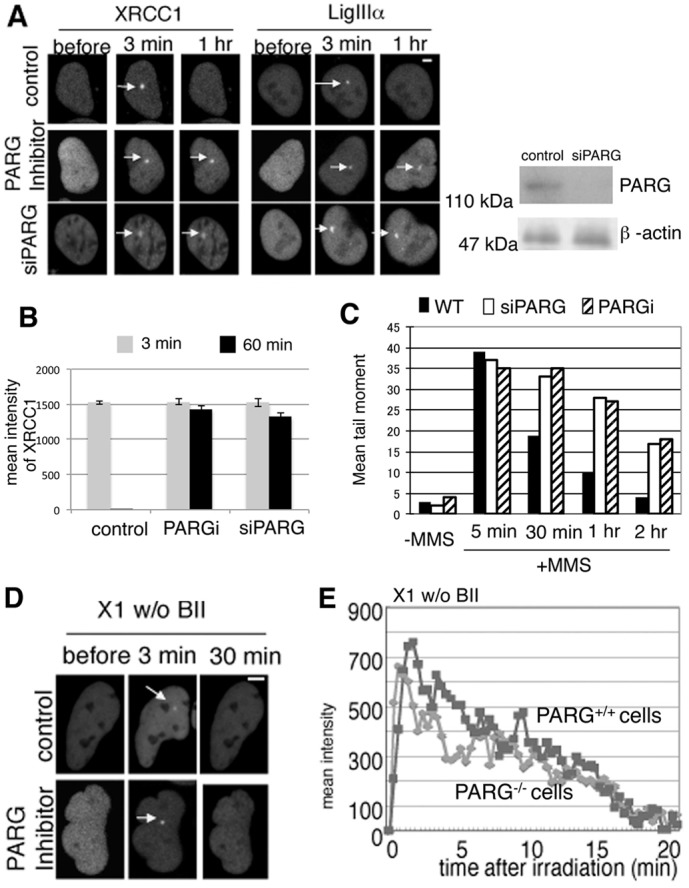
**PARG is necessary for translocation of XRCC1 and LigIIIα from pAR to SSBs.** (A) Accumulation and dissociation of XRCC1 and LigIIIα in siPARG- or PARG-inhibitor (100 µM tannic acid)-treated HeLa cells at the indicated time points after laser irradiation. Effects of siPARG in HeLa cells are shown in the right panel. (B) Quantification of the intensity of XRCC1 at sites of laser-induced DNA damage in siPARG- or PARG-inhibitor-treated HeLa cells 3 minutes and 1 hour after laser irradiation. (C) U2OS cells were treated with or without MMS and collected at the indicated times. Collected cells were used for the comet assay to analyze the remaining damage. The tail moments of 100 cells at 5 minutes, 30 minutes, 1 hour and 2 hours after treatment or without treatment were measured. (D) Accumulation and dissociation of X1w/oBII in HeLa cells 3 minutes and 30 minutes after treatment with 100 µM tannic acid PARG inhibitor. (E) Accumulation and dissociation of X1w/oBII in *Parg*^+/+^ (squares) and *Parg*^−/−^ (diamonds) cells at the indicated time points after laser irradiation. Arrows indicate the foci formed by the indicated proteins.

PARG accelerates SSBR (Fisher et al., 2007; Keil et al., 2006); PARG knockout mice are not viable, and PARG-deficient cells are sensitive to DNA damaging agents (Cortes et al., 2004). To understand the role of PARG in the dissociation of XRCC1 and LigIIIα, we measured the damage response of XRCC1 and LigIIIα under either siPARG or PARG inhibitor treatment. XRCC1 and LigIIIα were retained at sites of laser-induced SSBs for a longer time (Fig. 4A,B). The PARG inhibitor we used is active since it sensitized the cells to MMS (supplementary material Fig. S3A) and induced XRCC1 foci (Figs 1, 2). Moreover, we performed the comet assay to analyze the remaining damage after MMS treatment; 1 hour after the 40 µg/ml MMS treatment that we used for measuring foci formation of XRCC1 in Fig. 1, around 75% of the SSBs have been repaired. In addition, siPARG and PARGi treatment delayed the repair processes (Fig. 4C). We further confirmed the retention of XRCC1 and LigIIIα in *Parg*^−/−^ mouse ES cells compared with *Parg*^+/+^ mouse ES cells. Twenty minutes after irradiation, when XRCC1 had already dissociated from SSBs in wild-type ES cells, XRCC1 still remained at irradiated sites in *Parg*^−/−^ ES cells (supplementary material Fig. S3B), suggesting that XRCC1 cannot dissociate properly from pAR without PARG. Dissociation of GFP–LigIIIα was significantly delayed in *Parg*^−/−^ cells. Since XRCC1–LigIIIα forms a stable hetero-dimer complex (Caldecott et al., 1994), and the accumulation of LigIIIα is dependent on XRCC1 (Fig. 2C), retention of GFP–LigIIIα in PARG-suppressed cells at irradiated sites indicates that endogenous XRCC1 remains at pAR in PARG-suppressed cells. Importantly, X1w/oBII dissociated from SSBs as rapidly as in wild-type HeLa cells after treatment with the PARG inhibitor tannic acid, and in *Parg*^−/−^ cells (Fig. 4D,E). This indicates that the BRCTII domain is necessary for the retention of XRCC1 at damage sites in *Parg*^−/−^ ES cells as it can attach to sites of damage to prevent the release of XRCC1 from pAR. Thus, retention of XRCC1 at either pAR or damaged DNA seems to be dependent on the function of the BRCTII domain; therefore, both the BRCTII domain of XRCC1 and the degradation of pAR by PARG are necessary for the first translocation (from pAR to damage sites) of XRCC1 at an early stage of repair.

### Polyubiquitylation of XRCC1 requires efficient phosphorylation of XRCC1 in cells

**Fig. 5.**
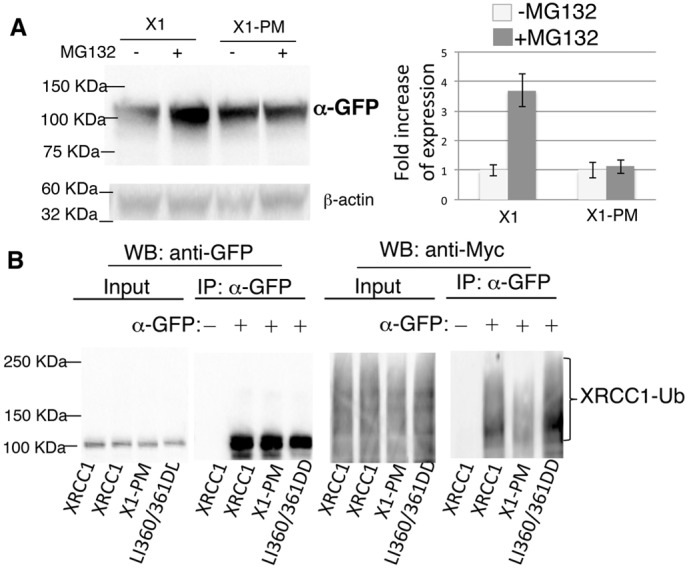
**Polyubiquitylation is regulated by polyADP-ribosylation and phosphorylation of XRCC1.** (A) Expression of XRCC1 or X1 pm in U2OS cells expressing GFP-tagged XRCC1 or X1 pm treated with MG132 for 30 minutes. β-actin was used as a loading control. (B) HEK 293 cells co-expressing myc–ubiquitin and GFP-tagged wild-type XRCC1, X1 pm or XRCC1 LL360/361DD were pulled down by anti-GFP and detected by anti-myc antibody.

After showing that PARG releases XRCC1 from pAR (first release) and the function of the BRCT II domain is to ‘fix’ XRCC1 at sites of damage, we investigated whether the dissociation of XRCC1 from sites of damage is just dissociation (second release) or occurs through protein degradation. XRCC1 has been shown to be a target for degradation by proteasomes with a ubiquitylation modification (Parsons et al., 2008). To learn whether the stability of X1 pm is affected by proteasome activity, cells expressing XRCC1 or X1 pm were treated with MG132. We found that expression of wild-type (WT) XRCC1 increased after MG132 treatment (Fig. 5A), indicating that polyubiquitylation of XRCC1 targets it for proteasome degradation. However, MG132 treatment did not alter the expression of X1 pm (Fig. 5A), indicating that X1 pm is stabilized at sites of damage in cells without proteasome degradation. The expression of WT XRCC1 and X1 pm with or without MG132 was quantified (Fig. 5A, right). The expression of WT XRCC1 increases 3- to 4-fold after MG132 treatment, whereas X1 pm expression was not altered. Therefore, phosphorylation of XRCC1 might be necessary for its ubiquitylation.

As we have shown, the BRCTII domain of XRCC1 targets XRCC1 for ubiquitylation in cells (Fig. 4E). We used two mutants of XRCC1 (XRCC1 LL360/361DD mutant, which abolishes pAR binding, and X1 pm, which abolishes its phosphorylation) to measure the effects of pARylation and phosphorylation on ubiquitylation in cells. Ubiquitylation of X1 pm was significantly decreased compared with wild-type XRCC1, but increased in the XRCC1LL360/361DD mutant (Fig. 5B). These results suggest that pARylation suppresses ubiquitylation of XRCC1, which might be necessary for stabilization of XRCC1 at pAR and at damage sites, whereas phosphorylation of XRCC1 is necessary for efficient ubiquitylation, which might facilitate the dissociation of XRCC1 from sites of SSBs at a late repair stage.

## Discussion

In this paper we have analyzed how XRCC1 and other SSBR proteins accumulate and dissociate at the sites of SSBs within cells and how the status of polyADP-ribosylation, phosphorylation and ubiquitylation contribute to the kinetics of XRCC1 itself and other repair factors. We show that a dynamic dissociation of XRCC1 is based on modifications of XRCC1 after pAR degradation. First, the BRCT II domain and function of PARG are necessary for the translocation of XRCC1 from pAR to sites of SSBs. Second, pARylation of XRCC1 prevents, but phosphorylation of XRCC1 is required, for efficient polyubiquitylation of XRCC1, therefore facilitating its degradation after repair completion.

### Translocation of XRCC1 from pAR to sites of SSBs depends on PARG and the BRCT II domain of XRCC1

One of our conclusions from this work is that the BRCT II domain of XRCC1 is necessary for the retention of XRCC1–LigIIIα and repair proteins such as Polβ at SSBs. X1w/oBII responds to local laser- and XPA-UVDE + local UVC-induced SSBs and shows very fast dissociation kinetics compared to those of wild-type XRCC1 (Figs 1, 2). We did not observe the damage response of X1w/oBII to MMS-induced damage (Fig. 1A). This is probably because MMS treats the whole cell and the induced damage is dispersed everywhere but it is not localized at restricted sites as with the other two methods, and also the kinetics of X1w/oBII movement are too fast to be observed.

Because the BRCT II domain interacts with LigIIIα, we reasoned that LigIIIα might be important for the retention of the XRCC1–LigIIIα complex at the damage site. Although we expected the LigIIIα Zn-finger or ligase domains to contribute to the retention, the BRCT domain of LigIIIα shows the same kinetics as that of the full-length LigIIIα (Fig. 3B). Therefore, it is the BRCT II domain of XRCC1 that determines the retention of the XRCC1–LigIIIα complex and other repair proteins at pAR. The crystal structure of the BRCT II domain of XRCC1 has been investigated and indicates how it plays a role in protein–protein interactions (Zhang et al., 1998). Of note, the activity of the BRCT II domain cannot replace the role of pAR as a sensor for damage because XRCC1 is mainly recruited to pAR. XRCC1 could not be recruited to sites of DNA damage although the purified XRCC1 protein shows some limited binding affinity for DNA *in vitro* (Mani et al., 2004; Nazarkina et al., 2007). Therefore, the BRCTII domain might contribute to this attachment process since XRCC1 is located beside the DNA within a certain physical distance as is the situation *in vitro*. More importantly, it is possible that the BRCTII domain might bind to specific chromatin proteins or to another DNA repair protein to be retained at the site of DNA damage. A recent study showed that the structural basis for forming either a homodimer or a heterodimer with LigIIIα and XRCC1-BRCTII is affected by the different linker regions of BRCT II (Cuneo et al., 2011), indicating that the role of the BRCT II domain could be more complicated as a LigIIIα partner.

PARG is a unique enzyme for degrading pAR, and SSBR is delayed in PARG-deficient cells (Fisher et al., 2007). There is evidence that ATP generated from pAR is necessary for the DNA ligation step, and XRCC1 interacts directly with PARG (Keil et al., 2006a; Oei and Ziegler, 2000). Without the degradation of pAR, most of the accumulated XRCC1–LigIIIα complex molecules are unable to dissociate from pAR, and the complex might be unable to approach the DNA to ligate it. Thus, PARG contributes to SSBR by degrading pAR, thereby promoting the attachment of the XRCC1–LigIIIα complex at the broken DNA. The retention of the XRCC1–LigIIIα complex at SSBs through XRCC1-BRCTII could also be coupled with a PARG-dependent ligation process (Keil et al., 2006a; Odell et al., 2011), and our data suggest that PARG plays an important role in molecular matchmaking of the repair complex organized by XRCC1.

### Proper dissociation kinetics of XRCC1 from SSBs requires its phosphorylation

It was reported that XRCC1-defective EM9 cells expressing X1 pm showed slow repair as measured by the comet assay (Loizou et al., 2004). We showed that X1 pm itself accumulated at SSBs as well as wild-type XRCC1, while PNK and APTX lost their ability for a damage response in X1 pm-expressing *Xrcc1*^−/−^ cells (Fig. 2D,F). X1 pm could recruit LigIIIα (Fig. 2B) but XRCC1 phosphorylation was also shown to be required for XRCC1–LigIIIα complex stability (Ström et al., 2011). These results suggest that the lack of recruitment of end processing enzymes PNK and APTX and the stability of the XRCC1–LigIIIα complex at SSBs could affect repair in X1 pm cells. A stronger affinity of this mutant for damaged DNA has been shown recently *in vitro* (Della-Maria et al., 2012; Ström et al., 2011), and this corresponds to our result that X1 pm forms foci after MMS treatment and is efficiently recruited to sites of damage in cells.

Interestingly, we showed that X1 pm is stable in cells and X1 pm is not efficiently ubiquitylated (Fig. 5). Recent studies showed involvement of nuclear proteasomes at DSBs, and the damage response of the nuclear proteasome activator PA28γ (REGgamma; PSME3) at sites of DSBs (Levy-Barda et al., 2011). PA28γ activates 11S as well as 20S proteasomes and is thought to be involved in repair processes in cell nuclei (Mao et al., 2008). We also transfected the nuclear proteasome activator PA28γ and found that PA28γ is located at sites of laser-induced DNA damage [supplementary material Fig. S4; and a previous study (Levy-Barda et al., 2011)]. In addition, RNF146, which is known to ubiquitylate XRCC1, is recruited to sites of laser-induced damage (Kang et al., 2011). These results indicate that proteasomes are activated at sites of laser-induced damage. Therefore, XRCC1 might be degraded at sites of damage by PA28γ-mediated proteasome degradation; this might be functional for the recycling proteins at the whole-cell level.

### XRCC1 dynamics after pAR degradation are regulated by its phosphorylation and ubiquitylation

**Fig. 6.**
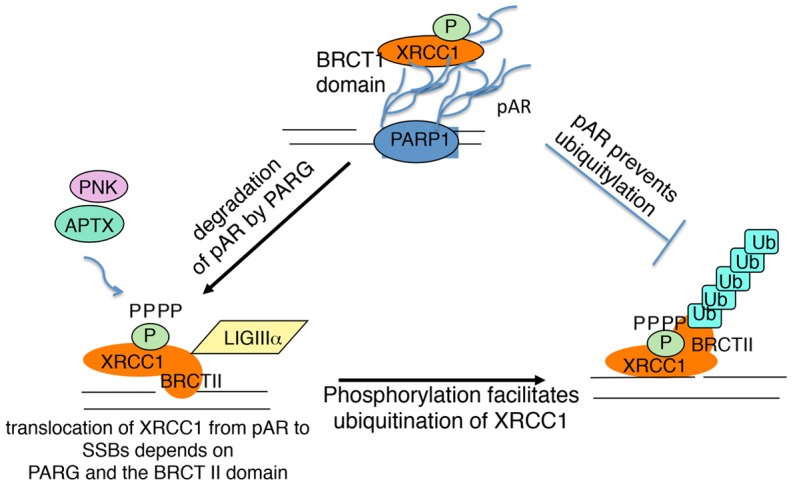
**A model of XRCC1-mediated repair of the SSB machinery.** The repair of SSBs within the cell proceeds with modifications of XRCC1. XRCC1 accumulates at pAR through its BRCT I domain, but will be retained at sites of SSBs through its BRCT II domain after pAR is degraded. Meanwhile, X1 pm shows an impaired ubiquitylation, indicating that phosphorylated XRCC1 is required for efficient ubiquitylation. Finally, XRCC1 might be ubiquitylated and degraded by proteasomes for protein recycling after repair completion. Thus, the repair of SSBs within the cell proceeds with formation and degradation of pAR in a manner that is dependent on other modifications and domains of XRCC1.

A model of translocation and degradation of XRCC1 after pAR degradation is shown in Fig. 6. Our data suggest that at the sites of SSBs, XRCC1 accumulates at pAR through its BRCT I domain, but XRCC1 will be retained at sites of SSBs through its BRCT II domain after pAR is degraded by PARG (Fig. 1). LigIIIα is not needed for XRCC1 retention but it is recruited to both pAR and SSBs through BRCT–BRCT-mediated interactions (Fig. 3). Although phosphorylation of XRCC1 is not required for recruitment of XRCC1 to pAR, it occurs in the early stage of the damage response and recruits the repair factors PNK and APTX to sites of SSBs (Figs 1, 2). Meanwhile, X1 pm showed an impaired ubiquitylation, indicating that phosphorylated XRCC1 is required for efficient ubiquitylation (Fig. 5). XRCC1-BRCTII forms either a homodimer or a heterodimer with LigIIIα, based on the different linker regions of BRCT II (Cuneo et al., 2011). Since the linker region adjacent to the BRCT II domain is targeted for phosphorylation and the BRCT II domain is targeted for ubiquitylation (Fig. 3E), the phosphorylation of XRCC1 might change the structure of the BRCT II domain so that it is exposed for ubiquitylation. This result supports our notion that the role of the BRCT II domain is more than that of a LigIIIα partner. Finally, XRCC1 might be ubiquitylated and degraded by proteasomes for protein recycling after repair completion. Thus, the repair of SSBs within the cell proceeds with formation and degradation of pAR in a manner that is dependent on other modifications and domains of XRCC1.

A scaffold protein such as XRCC1, with modifications and bound proteins, may be compared to a Swiss army knife with many tools. Studies of neural degenerative diseases demonstrate that the genesis of cerebellar interneurons requires XRCC1 (Lee et al., 2009). A spectrum of cancers, including ovarian, bladder, gastric, pancreatic, colorectal, skin and lung, have been reported with various XRCC1 polymorphisms (Cheng et al., 2012; Chiyomaru et al., 2012; Muñiz-Mendoza et al., 2012; Nakao et al., 2012; Wang et al., 2012; Zhi et al., 2012). A complete knockout of XRCC1 shows that it is essential for cell survival, and investigating the kinetics of polymorphisms of XRCC1 and their effects on the kinetics of other proteins used in this study would be useful for understanding the molecular mechanisms of tumorigenesis. This knowledge could also be useful for efficiently targeting the repair of SSBs at different stages regulated by distinct modifications, and could be utilized to inhibit the repair which otherwise lessens the effect of chemotherapy.

## Materials and Methods

### Plasmid construction for GFP-fused genes

Human cDNAs used in these experiments were obtained by amplification of HeLa cDNA by PCR. Amplified fragments with additional *Sal*I or *Xho*I sites at 5′ and *Not*I sites at 3′ ends were cloned into pEGFP-C1, -N1, or pDsRed-monomer-C1 vectors (Clontech). *YFP-PNK* (Loizou et al., 2004) and XRCC1 pm cDNA (Ser408Ala, Ser409Gly, Ser410Ala, Thr453Ala, Thr488Ala, Ser519Ala, Thr523Ala) were provided by Dr Caldecott (Loizou et al., 2004).

### Cell lines and transfections

The following cell lines were used: HeLa, U2OS, HEK 293, *Xrcc1*^+/+^ MEF (wild-type mouse embryonic fibroblast cell line), *Xrcc1*^−/−^ MEF, EM9 (*Xrcc1*^−/−^ Chinese hamster ovary cell line), and AA8 (parental CHO cell line of EM9). All the above cell lines were propagated in DMEM (Nissui) supplemented with 10% fetal bovine serum at 37°C, 5% CO_2_. Mouse J1U001 [*Parg*^+/+^ mouse embryonic stem (ES) cells] and 6D79-24 (*Parg*^−/−^ mouse ES cells) were grown in TX-WES (Cosmo Bio, Belgium) (Fujihara et al., 2009). Cells were plated in glass-bottomed dishes (Matsunami Glass) at 50% confluence 24 hours before transfection using Fugene-6 (Life Technology) or 96 hours before transfection using an siRNA transfection kit (Life Technology) and irradiated with a 405 nm laser-light under a microscope 48 hours after transfection. siRNA for the LigIIIα sense sequence is 5′-GCAUCAUCAGGUUGAUCAAAC-3′.

### Microscopy and laser-light irradiation

Fluorescence images were obtained and processed using an FV-500 confocal scanning laser microscopy system (Olympus). The power of the 405 nm laser (original 50 mW) was adjusted with time at a final output of 5 mW after passing through the lens. 10 mseconds of 405 laser light irradiation mainly produces SSBs in the cell. Cells were incubated with Opti-MEM (Gibco) in glass-bottomed dishes, which were covered with microscopy live-cell imaging chambers (Olympus) to prevent evaporation on the 37°C heating plate. The mean intensity of the focus was obtained after subtraction of the background intensity in the irradiated cell. For quantification of cell fractions with MMS-induced foci, BrdU incorporation and XPA-UVDE foci, a total of 100 cells were counted in each experiment. To measure the damage response to laser micro-irradiation, in each experiment, damage response in over thirty cells was examined. Each experiment was performed three times. The data are presented as means and standard deviations (s.d.); *n* = 10 cells.

### XPA-UVDE, local irradiation and comet assay

Local UV irradiation of XPA-UVDE cells was performed as described previously. Cells in 35-mm glass-bottomed culture dishes (poly-D-lysine coated; MatTek) were covered with a polycarbonate isopore membrane filter with pores of 3 µm in diameter (Millipore) and irradiated with 100 J/m^2^ UVC. SSBs were quantified using an alkali comet assay protocol (Comet Assay Kit, Trevigen). After treatment with 40 µg/ml MMS (Wako) for 20 minutes in DMEM, cells were washed with 1× PBS and incubated in fresh DMEM for the time indicated. Average tail moments from 100 cells per sample were obtained using Comet Assay IV software (Perceptive Instruments), and data are shown as mean values from three independent experiments.

### Purification of the BRCT II domain of XRCC1

The XRCC1 BRCT II domain was subcloned into a pet 16-N His tag vector. The expressed protein in *E. coli* was purified through an Ni-NTA column (Qiagen). BRCT II proteins were eluted with 500 µM imidazole. The elution fraction was passed through a heparin column (Bio-Rad) followed by a Q FF column (GE Healthcare Life Sciences). After elution from the column, it was finally purified by gel filtration.

### Gel shift assay

The gel shift assay followed the Promega protocol. In short, the indicated amount of purified protein was incubated with 0.5 pmol ^32^P-labeled nicked DNA at room temperature for 30 minutes. After incubation, the solution was run on a nondenaturing, 4% acrylamide gel in 0.5× TBE buffer.

### Chemicals

A final concentration of 40 µg/ml methyl-methane sulfonate (MMS; Sigma) was added to PBS for 20 minutes incubation. A final concentration of 4 µM of the PARP inhibitor PJ34 (Sigma) or 100 nM olaparib (Sigma) was added to the medium for 30 minutes. A final concentration of 100 µM tannic acid (Sigma) was used as a PARG inhibitor. 10 µM MG132 (Cal Biochem) was added to medium for 30 minutes.

### Immunostaining, Immunoprecipitation and western blot assays

HEK 293 cells were co-transfected with GFP-tagged wild-type XRCC1 or its mutant and myc-ubiquitin for immunoprecipitation. Two days after transfection, cell lysates were prepared in 1 ml of lysis buffer (10 mM Hepes, pH 7.6, 250 mM NaCl, 0.1% Nonidet P-40, 5 mM EDTA, 1 mM phenylmethylsulfonyl fluoride). For immunoprecipitation, 1 µg anti-GFP monoclonal antibody (Roche, 11 814 460 001), and 30 µl of protein-G–Sepharose beads (Amersham Biosciences) were added to each lysate. Mixtures were incubated at 4°C for 4 hours with rotation, the supernatant was removed, and protein beads were washed three times using 0.4 ml of lysis buffer. For immunostaining, cells in a medium for immunostaining were fixed with methanol:acetone (1∶1) for 10 minutes at −20°C. The fixed cells were dried, then rinsed once with PBS and incubated in blocking buffer (PBS containing the blocking reagent NEN) at 30°C for 30 minutes. Cells were then incubated with the first antibody overnight. Cells were washed three times with PBST (PBS with Tween 20) buffer and incubated with Alexa Fluor 488 donkey anti-goat immunoglobulin G conjugate or Alexa Fluor 488 donkey anti-rabbit immunoglobulin G conjugate. Cell samples were then mounted in drops of PermaFluor (Immunon). For western blotting, whole-cell extracts were applied to sodium dodecyl sulfate polyacrylamide gels, and transferred to nitrocellulose membranes. Membranes were blocked for 1 hour in Tris-buffered saline-Tween 20 (TTBS) containing 5% skimmed milk (blocking buffer) and then incubated with antibody at the dilution condition recommended by the manufacturer in blocking buffer at 4°C. Membranes were washed in TTBS and incubated in horseradish peroxidase-conjugated anti-rabbit immunoglobulin G (IgG; Santa Cruz) as appropriate, at a 1∶3000 dilution for 30 minutes at room temperature. Membranes were then washed with PBST and antibody complexes were detected by enhanced chemiluminescence (Amersham). Antibodies used were: anti-poly(ADP-ribose) (BD Pharmingen, 51-8114KC), anti-GFP (Roche, 11814460001), anti-β-actin (sc-1616, Santa Cruz) and c-Myc (Santa-Cruz, SC-789) at the dilution recommended by the manufacturer, in blocking buffer overnight at 4°C. Membranes were washed in TTBS and incubated in horseradish-peroxidase-conjugated anti-rabbit IgG (Santa Cruz) as appropriate, at a 1∶3000 dilution for 30 minutes at room temperature. Membranes were then washed with PBST, and antibody complexes were detected by enhanced chemiluminescence (Amersham).

## Supplementary Material

Supplementary Material
