# Supplementary material for: Damage response of XRCC1 at sites of DNA single strand breaks is regulated by phosphorylation and ubiquitylation after degradation of poly(ADP-ribose)
Source: J Cell Sci. 2013 Oct 1;126(19):4414–23. doi: 10.1242/jcs.128272 (PMC3784821; doi:10.1242/jcs.128272)
Supplement: Supplementary Material [file supp_126_19_4414__index.html]

Supplementary Material 

# Damage response of XRCC1 at sites of DNA single strand breaks is regulated by phosphorylation and ubiquitylation after degradation of poly(ADP-ribose)

## JCS128272 Supplementary Material

**Files in this Data Supplement:**

- **Supplementary Material PDF**
