# Supplementary material for: Damage response of XRCC1 at sites of DNA single strand breaks is regulated by phosphorylation and ubiquitylation after degradation of poly(ADP-ribose)
Source: J Cell Sci. 2013 Oct 1;126(19):4414–23. doi: 10.1242/jcs.128272 (PMC3784821; doi:10.1242/jcs.128272)
Supplement: Supplementary Material [file supp_jcs.128272_JCS128272.pdf]

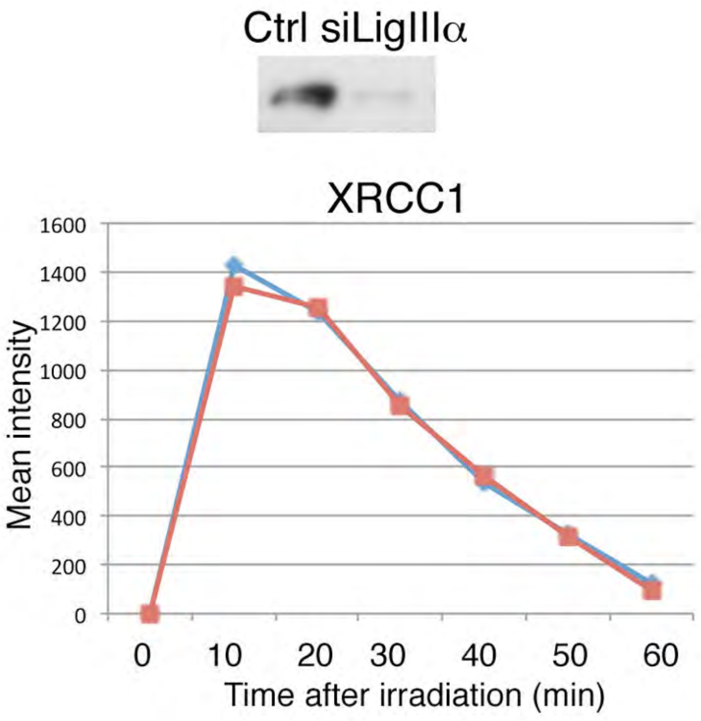

Fig. S1. siLigIIIα does not affect the repair kinetics of XRCC1. Kinetics of XRCC1 in U2OS cells after laser irradiation with (blue) or without (red) siRNA for LigIIIα.

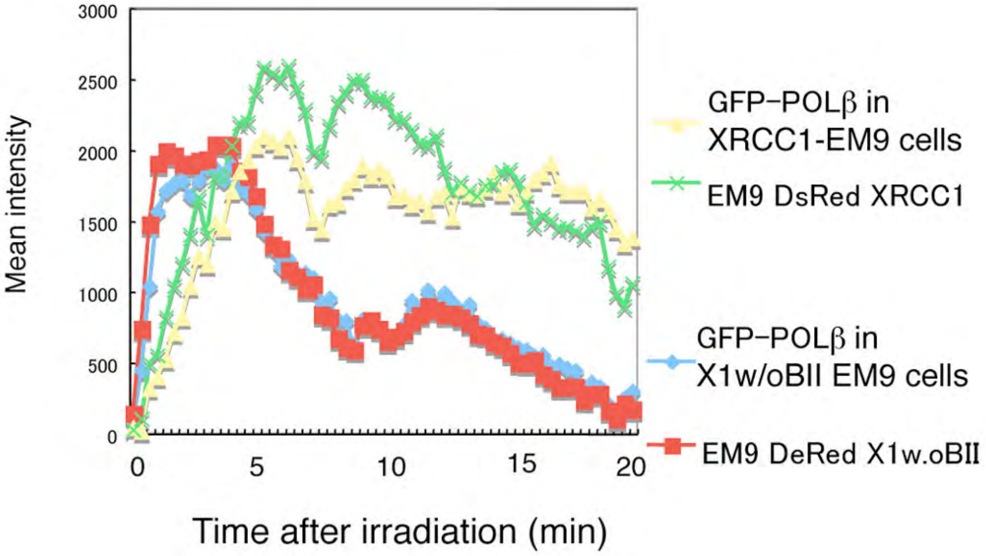

Fig. S2. Retention of Polβ at sites of SSBs is dependent on XRCC1-BRCT II. Kinetics of Polβ in EM9 cells expressing full-length XRCC1 (yellow) or X1w/oBII (blue). Kinetics of full-length XRCC1 (green) and X1w/oBII in EM9 (red) cells are shown as well.

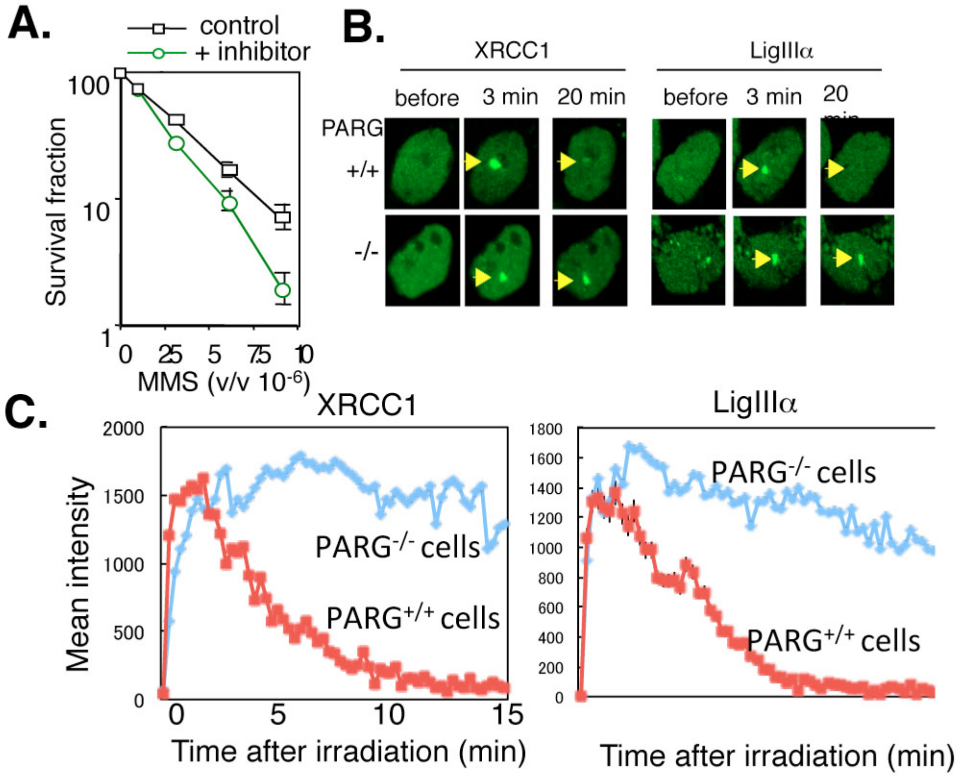

Fig. S3. Effects of PARG on kinetics of XRCC1 and LigIIIα. A. HeLa cell survival curve after treatment with the PARG inhibitor tannic acid at the indicated dose. **B.** Accumulation of GFP-XRCC1 and -LigIIIα in $PARG^{+/+}$ and $PARG^{-/-}$ cells 3 min and 20 min after UVA laser irradiation. **C.** Kinetics of accumulation of GFP-XRCC1 and -LigIIIα in $PARG^{+/+}$ and $PARG^{-/-}$ cells after laser irradiation.

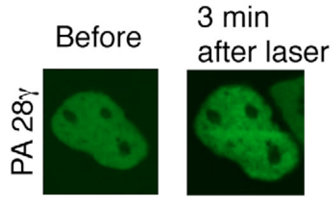

Fig. S4. Recruitment of proteasomes at sites of DNA damage. PA28γ responds to DNA damage induced by laser.
